# The magnitude and mechanisms of the weekend effect in hospital admissions: A protocol for a mixed methods review incorporating a systematic review and framework synthesis

**DOI:** 10.1186/s13643-016-0260-2

**Published:** 2016-05-21

**Authors:** Yen-Fu Chen, Amunpreet Boyal, Elizabeth Sutton, Xavier Armoiry, Samuel Watson, Julian Bion, Carolyn Tarrant, on behalf of the HiSLAC Collaborative

**Affiliations:** Division of Health Sciences, Warwick Medical School, University of Warwick, Coventry, CV4 7AL UK; Queen Elizabeth Hospital Birmingham and University of Birmingham, Birmingham, UK; Department of Health Sciences, University of Leicester, Leicester, UK; Warwick Centre for Applied Health Research & Delivery, Division of Health Sciences, Warwick Medical School, University of Warwick, Coventry, CV4 7AL UK

**Keywords:** Weekend, Mortality, Hospital admission, Hospitalization, Secondary care, Delivery of health care, Health services research, Health care evaluation mechanisms, Quality of health care, Risk adjustment

## Abstract

**Background:**

Growing literature has demonstrated that patients admitted to hospital during weekends tend to have less favourable outcomes, including increased mortality, compared with similar patients admitted during weekdays. Major policy interventions such as the 7-day services programme in the UK NHS have been initiated to reduce this weekend effect, although the mechanisms behind the effect are unclear. Here, we propose a mixed methods review to systematically examine the literature surrounding the magnitude and mechanisms of the weekend effect.

**Methods:**

MEDLINE, CINAHL, HMIC, EMBASE, EthOS, CPCI and the Cochrane Library were searched from Jan 2000 to April 2015 using terms related to ‘weekends or out-of-hours’ and ‘hospital admissions’. The 5404 retrieved records were screened by the review team, and will feed into two component reviews: a systematic review of the magnitude of the weekend effect and a framework synthesis of the mechanisms of the weekend effect. A repeat search of MEDLINE will be conducted mid-2016 to update both component reviews. The systematic review will include quantitative studies of non-specific hospital admissions. The primary outcome is the weekend effect on mortality, which will be estimated using a Bayesian random effects meta-analysis. Weekend effects on adverse events, length of hospital stay and patient experience will also be examined. The development of the framework synthesis has been informed by the initial scoping of the literature and focus group discussions. The synthesis will examine both quantitative and qualitative studies that have compared the processes and quality of care between weekends and weekdays, and explicate the underlying mechanisms of the weekend effect.

**Discussion:**

The weekend effect is a complex phenomenon that has major implications for the organisation of health services. Its magnitude and underlying mechanisms have been subject to heated debate. Published literature reviews have adopted restricted scopes or methods and mainly focused on quantitative evidence. This proposed review intends to provide a comprehensive and in-depth synthesis of diverse evidence to inform future policy and research aiming to address the weekend effect.

**Systematic review registration:**

PROSPERO 2016: CRD42016036487

**Electronic supplementary material:**

The online version of this article (doi:10.1186/s13643-016-0260-2) contains supplementary material, which is available to authorized users.

## Background

A broad literature identifies that weekend admission to hospital, whether emergency [1, 2] or elective [3, 4], is associated with increased mortality. The impact on mortality is deferred—it occurs some days after weekend admission [5]. The cause for this effect is unclear, but is likely to be mediated partly through difference in case mix between weekdays and weekends and in part through different structures and processes of care at weekends, including the intensity of specialist provision and support services at weekends [6]. In the United Kingdom (UK), the National Health Service (NHS) has launched the 7-day services programme to create consistent quality of and access to health care throughout the week [7]. Quantifying the magnitude of the effect and understanding the underlying mechanisms by which improvements in patient outcomes might be achieved is important for justifying the investment required, and in differentiating effective interventions from secular trends.

The literature on the weekend effect is diffuse and rapidly growing [1, 2, 8–11]. However, existing literature reviews have been limited by a focus on specific conditions and patient populations [8–10], have not included quantitative synthesis [1, 2] or have lacked a detailed account of methodology [11]; none have systematically examined qualitative evidence. Given the ongoing controversies related to the subject and the need to have a better understanding of both the magnitude and mechanisms of the weekend effect to inform the planning, implementation and evaluation of any interventions and service re-design to tackle this issue, we will undertake a mixed methods review [12] of the literature as part of the High-intensity Specialist Led Acute Care (HiSLAC) Project, which brings together a broadly based coalition of patients, clinicians, researchers and policy-makers across the NHS in England to evaluate the impact of changes in specialist provision arising from NHS England’s 7-day services programme on the weekend effect. This protocol provides an overview of our methodological approach and detailed plan for the mixed methods review. The preparation of the protocol conforms to the PRISMA-P guidelines [13], and a completed PRISMA-P checklist can be found online [see Additional file 1].

## Method/design

The mixed methods review will include two complementary components: first, quantitative evidence will be synthesised to confirm the magnitude of the weekend effect and to identify possible moderators and mediators of the effect via a systematic review that incorporates meta-analyses. Second, we will use framework synthesis methodology [14, 15] to explore both qualitative and quantitative evidence on the mechanisms of the weekend effect.

### Research question and aims

The mixed methods review seeks to answer a broad research question:

*What is the magnitude of the weekend effect associated with hospital admissions, and what are the likely mechanisms through which differences in structure and process of care between weekdays and weekends contribute to this effect?*

For this review, we define the weekend effect as differences in quality of care and patient outcomes between weekend and weekday admissions. While we mainly focus on mortality, in seeking to understand the underlying mechanisms behind the weekend effect we will also characterise differences between weekend and weekday care in relation to length of stay, patient experience and quality of care. The broad research question will be addressed through a systematic review and a framework synthesis with complementary aims and methodological approaches as described below.

1. The systematic review will focus on quantitative evidence and aims to:
  Estimate the magnitude of the weekend effect on mortality, adverse events, length of hospital stay and quantitatively measured patient experience.
  Quantitatively explore potential mediators and contextual modifiers of the weekend effect.
2. The framework synthesis will examine both qualitative and quantitative evidence on the mechanisms, and contextual modifiers of the weekend effect. The review will explore how differences in structure (e.g. staffing levels), care processes (e.g. the nature of routine patient review, waiting times for procedures), and case mix between weekend and weekday, impact on quality of care and patient outcomes [16]. We will take a broad definition of quality of care, drawing on Darzi’s three elements of quality [17] (see Table 1); this definition has become widely accepted as a shared definition of quality within the NHS [18].

**Table 1:** Definition of quality of care used to inform the framework synthesis

Effectiveness of the treatment and care provided to patients (e.g. process-related measures including adherence to guidelines; clinical outcomes; patient‐related outcomes)	
The safety of treatment and care provided to patients (e.g. omissions in care; delays; medical errors; adverse events)	
The experience patients (and staff) have of treatment and care

### Approach

**Fig. 1:**
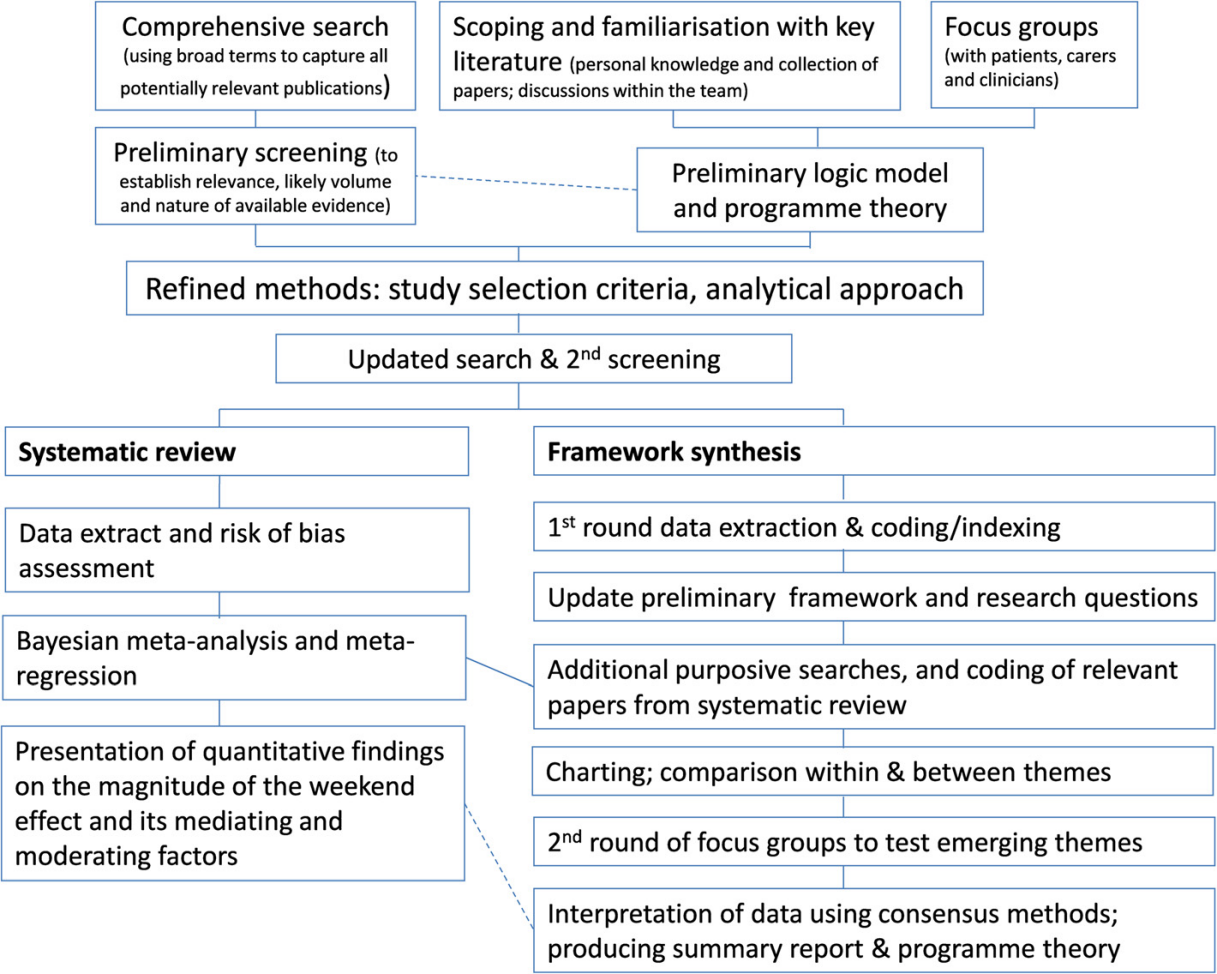
Overall approach for the mixed methods review

We have adopted a multi-stage, iterative approach in order to cover the most relevant literature (which is large and diverse) to answer the research question within the available resources, and to ensure that the two components of the review are closely linked with and complement each other throughout the review process. Figure 1 illustrates the overall approach that we have adopted. First, we undertook a comprehensive and systematic literature search to establish the potential volume and nature of available evidence. Initial scoping of this literature, along with focus groups with staff and patients, informed the development of the methodological approach for the two component reviews.

### Literature search

In order to scope the diverse literature related to the weekend effect, we searched bibliographic databases including MEDLINE, CINAHL, HMIC, EMBASE, EThOS, CPCI (Conference Proceedings Citation Index) and the Cochrane Library in April 2015 to look for material published from 2000 onwards. The date was chosen in view of the fact that the weekend effect was first highlighted in a seminal paper published in 2001 [19]. Following iterations of pilot searches, we used a search strategy that combined terms relating to ‘weekend/weekday or out-of-hours’, and to ‘hospital admissions’. The key words and index terms used are shown in Appendix 1 [see Additional file 2]. The retrieved records were imported into EndNote (Thomson Reuters) and duplicate records were removed. This initial comprehensive search retrieved 5404 unique references, which underwent preliminary screening by the review team to evaluate their potential relevance to the systematic review and framework synthesis. An updated search will be carried out in MEDLINE in mid-2016 to capture recent papers published since our initial search. Only MEDLINE will be searched as our initial screening indicates that the vast majority of relevant publications are covered by MEDLINE. Reference lists of included studies will be checked to identify further literature, and experts will be consulted through the HiSLAC project to ensure that no crucial evidence is missed. Additional purposive searches will be carried out at the second stage of the framework synthesis (described below).

### Specification of component reviews

**Fig. 2:**
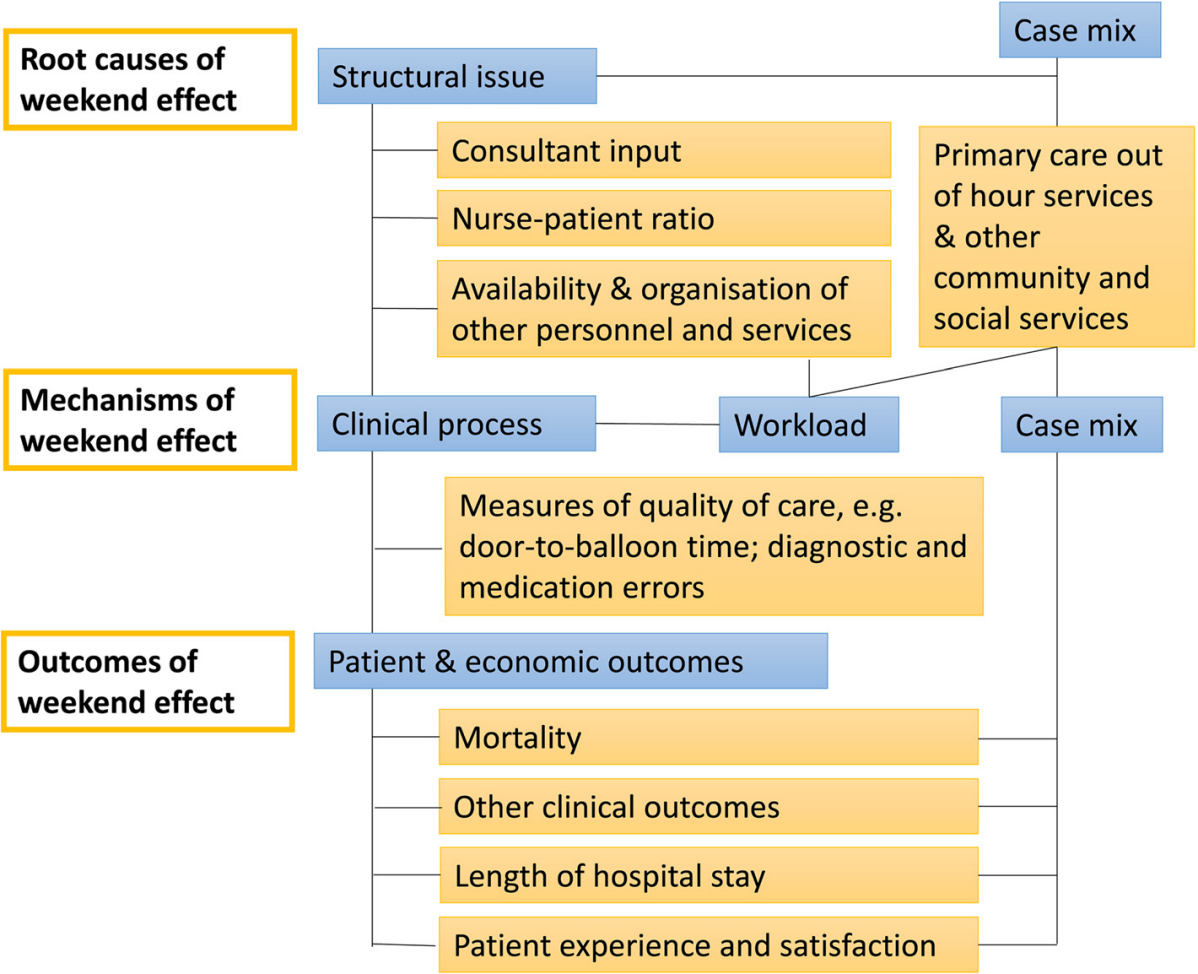
Preliminary logic model for the mixed methods review on weekend effect

Initial scoping of identified literature along with findings from focus groups contributed to the development of a preliminary logic model of the weekend effect that informs the design of the literature reviews. We conducted two focus groups in a single NHS Trust, with eight healthcare staff and seven patients and relatives. Staff were purposively sampled to represent a range of roles involved in the care of patients admitted as emergencies at the weekend (including consultants, nurses and junior doctors); patients were identified through a local acute care patient group. Focus groups explored experiences of the differences between weekday and weekend care, views on the factors that are associated with an increased mortality risk, and views of the mechanisms through which this occurred. Group discussions were recorded, transcribed verbatim and analysed using a thematic approach [20]. The preliminary logic model was revised iteratively through discussion with experts on the study steering and advisory groups. The model is shown in Fig. 2. This model has been used to focus and refine the screening approach and inclusion criteria for each component review, and to provide a source of themes to structure analysis in the framework synthesis.

**Fig. 3:**
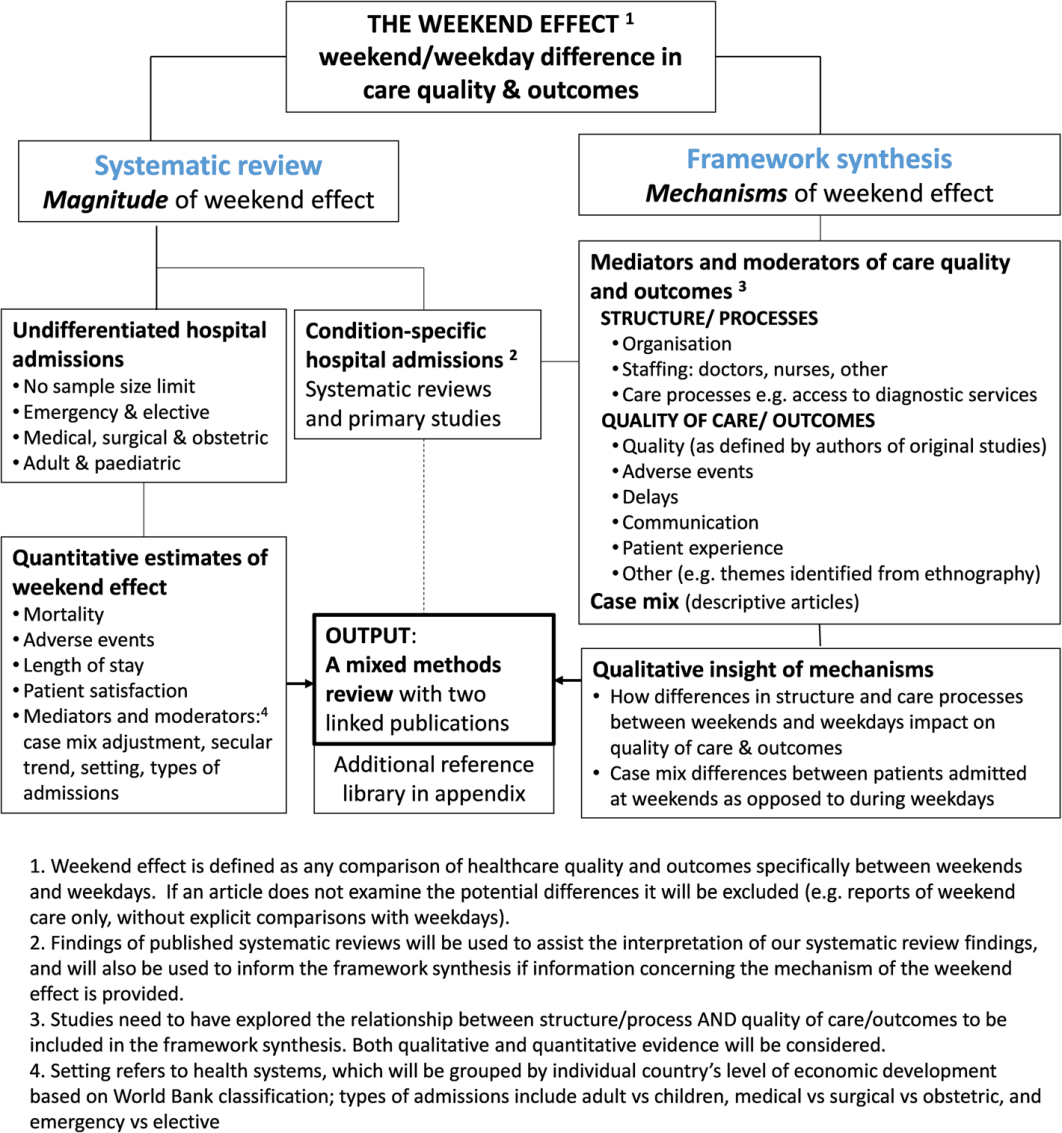
Scope and literature covered by the two components of the mixed methods review

Figure 3 shows the approach that will be used to screen papers identified through the systematic search for inclusion in the systematic review and/or the framework synthesis. Appendix 2 [see Additional file 2] shows the screening form to be used. The proposed screening and analysis processes for the two component reviews are described in more detail below.

### Methods for the systematic review

#### Study selection

Quantitative study of any design will be considered. To be included in the systematic review, a study needs to meet the following criteria:

1. Undifferentiated admissions to acute hospitals, i.e. admissions across different conditions or specialties rather than admissions related to specific conditions or specialties. Non-specific admissions related to adults and/or children, medical, surgical and/or obstetric specialties, and emergency and/or elective admissions are all eligible. Studies that reported both aggregated and condition-specific weekend effects will be included but only the aggregated data will be included in the quantitative analysis of the systematic review. Condition-specific data will be considered by framework synthesis. Our decision to focus on unselected admissions and to leave out condition-specific admissions from the systematic review was mainly pragmatic given our time and resource constraint and the large number of published studies on the weekend effect for condition-specific admissions. In addition we became aware of the publication of a systematic review and meta-analysis focusing on condition-specific admissions during the preparation of this protocol [21]. This obliterates the need for undertaking similar quantitative analyses which would be an unnecessary duplication.
2. Compared the following outcomes of interest between weekend admissions with weekday admissions, or between patients having their critical period of care at weekends (e.g. receiving a surgical procedure just before weekend; giving birth during weekend) with those having their critical period of care on weekdays: mortality, adverse events (defined as any undesirable events caused by medical management rather than the underlying condition of the patient), length of hospital day and quantitatively measured patient satisfaction.

The full-text article or report of the study needs to be available. Studies that are only available as conference abstracts (despite an attempt to request full-text reports or additional information from study authors) will be excluded. No language restriction will be applied. For studies published in non-English language, we will try to obtain relevant information from the authors or obtain translations. A list will be provided for studies which we are not able to include due to insufficient information despite these efforts.

While weekends normally refer to Saturdays and Sundays, alternative definitions of weekends in accordance with cultural and religious variations will be accepted, provided that the defined weekends are the regular dates devoted to rest, during which the level of staffing is expected to be reduced unless a special arrangement is made. Studies in which outcomes were compared between out-of-hours and regular hours will be included if the out-of-hours include weekends. Studies which exclusively compared daytime versus night-time will be excluded.

Study selection will be carried out independently by two reviewers, with any discrepancies resolved by discussion or being referred to the wider review team for discussion and arbitration. Decisions with regard to study selection with relevant notes from discussions will be recorded in a spreadsheet.

#### Data extraction

Key characteristics of included studies will be extracted and coded by one reviewer and independently checked by another reviewer in a structured spreadsheet. Data to be extracted include:

1. Setting: country, study period, data source, type of data (cross-sectional or longitudinal), type of admission (all, emergency or elective).
2. Patient population: type of patients (all, adults or children), type of procedures (all, medical or surgical or obstetric).
3. Inclusion/exclusion criteria; statement concerning completeness and/or accuracy of data.
4. Study design and sample size.
5. Comparisons made (weekends vs. weekdays; our-of-hours vs. regular working hours); definitions of weekdays and weekends; reference day(s) for the comparison and rationale for the choice.
6. Methods for estimating the weekend effect, including statistical methods and variables (e.g. patient case-mix and hospital characteristics) included in statistical adjustments.
7. Outcome measures and their definitions: mortality (e.g. in-hospital, 7-day etc.), adverse events, length of hospital stay, and quantitatively measured patient satisfaction (e.g. NHS Friends and Family Test (FFT).
8. Reported weekend effects: these are expressed as risk/rate ratios, odds ratios or hazard ratios (for mortality) and differences in means or medians. Both adjusted and unadjusted values along with their confidence interval/standard errors will be recorded.
9. Results of any sensitivity analyses performed.

Data extraction for the systematic review will be conducted by a team. A training workshop will be provided, led by YFC, who is highly experienced in conducting systematic reviews. Discrepancies in extracted data and coding decision will be resolved by discussion or seeking further advice from the research team. Study authors will be contacted for clarification and/or for additional information if needed. Papers screened for the systematic review will also be assessed against the criteria for the framework synthesis and, if appropriate, passed to the team leading this component of the review.

#### Risk of bias assessment

Assessment of risk of bias of included studies at study level will be based on the Newcastle-Ottawa quality assessment scale for cohort studies (and case-control studies if studies of this design were found) [22]. The tool will be piloted on a number of included studies during the data extraction training workshop with subsequent modifications if necessary to ensure that reviewers have common understanding of how to rate important items potentially associated with bias, which include:

1. Quality of data (completeness and accuracy of coding).
2. Definitions of weekday and weekend admissions.
3. Comparability of patients admitted during weekdays and during weekends.
4. Adequacy of statistical (including case-mix) adjustment—this will be classified as adequate adjustment (adjusted for measures reflecting the frailty of the patient, such as physiological and/or biochemical measures, and all other major potential confounders such as age, diagnosis and co-morbidity), partial adjustment (adjusted for all major potential confounders except measures of frailty) or inadequate adjustment (one or more major confounders were not adjusted for).

While all relevant items will be assessed to uncover crucial methodological issues, we will focus in particular on adequacy of statistical adjustment, which will be used to inform sensitivity analysis (described below). Risk of bias assessment will be undertaken independently by two members of the review team and any discrepancies will be resolved through discussion or seeking further advice from the research team. Results of risk of bias assessment will be finalised before data analysis takes place.

#### Quantitative estimation of weekend effects

Results from the included studies will be meta-analysed using a Bayesian random effects model that allows for within study variation and between study heterogeneity. Analyses will be conducted with STAN [23]. Our primary outcome is mortality as it is an objective and important outcome that has triggered policy changes. The primary analysis will be undertaken using (log) adjusted odds ratios. Where multiple measures of mortality are reported (e.g. in-hospital mortality, 30-day mortality etc.), in-hospital mortality will be used in the primary analysis. Where multiple estimates based on different reference day(s) were reported, we will use the estimate based on Wednesday (or the period including Wednesday) being the reference group. Heterogeneity between studies will be assessed using the *I*^2^ statistic (with 50 % or more indicating substantial heterogeneity). If studies are identified that provide multiple estimates from different samples (e.g. by time period) we will incorporate the third level in the model to allow for within sample variation.

Where different studies appear to have used data from the same data source and covered the same period (or an overlapping period), the studies will be assessed by the following criteria in order to select the most appropriate data for meta-analysis: (1) best quality in terms of adjustment for potential confounding factors, (2) largest sample size and (3) most up to date.

#### Exploring potential sources of heterogeneity

We will investigate whether there is evidence that the weekend effect has changed over time in a secondary analysis. Models with different specifications for time (e.g. linear time trend, quadratic function) will be estimated and we will select the best fitting model using the Watanabe Akaike Information Criterion (WAIC).

If there is further substantial heterogeneity between studies, we will conduct an exploratory meta-regression provided there is a sufficient number of studies. The following variables (explanatory factors which could be either moderators or mediators of weekend effects) are specified a priori:

- Adequacy of case-mix adjustment (adequate, partial, inadequate)
- Time
- Population: all vs. adults vs. children
- Type of admissions: all vs. medical vs. surgical vs. obstetric
- Urgency of admissions: all vs. emergency vs. elective
- Country category (high, upper-middle, lower-middle and low income based on the World Bank [24]

Results of additional subgroup analysis from individual studies (with the subgroup defined by a variable other than those listed above) will also be recorded. If a high level of between-study heterogeneity remains unexplained by the pre-specified variables included in the meta-regression, additional variables may be explored in further subgroup analyses/meta-regression (e.g. if based on observed data it was suspected that the weekend effect varies by country within an income category). These will be clearly described as post hoc, exploratory analyses.

Sensitivity analysis will be carried out to assess the robustness of primary analysis on mortality:

1. Comparison between studies that are considered to have inadequate case-mix adjustment to those with adequate adjustment, determined during risk of bias assessment prior to the commencement of data analysis.
2. Comparison between studies that compared weekends vs. weekdays or out-of-hours vs. regular hours.
3. Use of alternative measures of mortality (e.g. 7-day, 30-day or longer).
4. Use of data based on alternative reference day(s) as the reference group for studies that reported multiple sets of estimates.
5. If sufficient data is available, meta-analysis of ratios of adjusted vs unadjusted weekend effects will also be carried out to illustrate the potential impact of risk adjustment.

#### Assessment of publication bias

We will construct funnel plots to assess small study effects, for which publication bias and outcome reporting bias are among the possible causes [25]. We will interpret the funnel plots based on both visual inspection of the plots and other information concerning the clinical and methodological heterogeneity between studies. If there is evidence of publication bias, we will use a data augmentation approach to derive an unbiased pooled estimator [26].

#### Presentation of findings and assessment of overall quality of evidence

The findings of the systematic review will be presented alongside findings of the framework synthesis as linked papers. The systematic review will be reported in accordance with the Meta-analysis of Observational Studies in Epidemiology (MOOSE) guideline [27]. Summary tables of study characteristics and risk of bias assessment for individual studies will be provided. Results of meta-analyses will be presented in forest plots and/or tabulated. The overall quality of evidence across studies will be assessed on the basis of the GRADE approach, which takes into account risk of bias in individual studies, bias across studies, precision of estimates and consistency of evidence.

### Methods for the framework synthesis

#### Study selection

Both studies that include undifferentiated hospital admissions, and studies that focus on defined patient populations, will be candidates for inclusion. Quantitative and qualitative studies will be included. A study needs to meet both of the following criteria to be included:

1. Includes data on structure or process of care at the weekend as compared to weekdays
2. Includes data on quality of care or outcomes associated with these differences in care provision

We will also include papers that contain data on case mix differences between patients admitted at the weekend as compared to on a weekday.

The full-text article or report of the study needs to be available. Studies that are only available as conference abstracts (despite an attempt to request full-text reports from study authors) will be excluded.

Relevance will take precedence to quality to avoid discounting studies that may make an important contribution to the narrative. However, papers deemed to be ‘fatally flawed’ will be screened out based on criteria proposed by Dixon-Woods et al, 2011 [14] (see Table 2). Study selection will be carried out independently by two reviewers with uncertainty resolved by discussions.

**Table 2:** Appraisal prompts for informing judgements about quality of papers

Are the aims and objectives of the research clearly stated?	
Is the research design clearly specified and appropriate for the aims and objectives of the research?	
Do the researchers provide a clear account of the process by which their findings were produced?	
Do the researchers display enough data to support their interpretations and conclusions?	
Is the method of analysis appropriate and adequately explicated?	

Reproduced from: Dixon-Woods et al. *BMC Medical Research Methodology* 2006;6:35 doi:10.1186/1471-2288-6-35

#### Data extraction

Data extraction will be conducted using a simple coding frame including: study aims, study context, participants, methods of data collection and analysis, key findings. Initial data extraction will be undertaken by one reviewer and this will be checked by another review to ensure consistency.

#### Synthesis and interpretation

The process of synthesis will involve initial familiarisation with the data. The preliminary logic model will be used as a framework to organise findings from the literature, aided by NVivo 10. Papers will be initially index coded. Framework matrix coding will then be used to summarise data into charts organised around key differences between weekend and weekday care, drawing out the evidence for underlying mechanisms (see example in Table 3). This approach will allow us to integrate and interpret qualitative evidence with evidence from studies using quantitative or mixed methods.

**Table 3:** Example of charting

Chart 1: Staffing	1.1 Which staff, why	1.2 Staff levels	1.3 Role in preventing weekend effect	1.4 Views on safe levels of staffing	1.5 Impact of low staffing levels
Case 1 (reference)	Nurses—page no.	Ratios for safe care at weekends	Act as key knowledge keeper—vital in handover of information on discharge	Needs to be more ….	
Case 2 (reference)	Doctors	Rotas	Trainees tend to be more in evidence at weekends = lack of expertise available		Patients rushed to intensive care as no one to provide review when required
Case 3 (Reference)	Phlebotomists	Nonexistent	Delays in handover of care and discharge	Rotas need adjusting to include more staff	

Data extraction and synthesis will be conducted as a multi-step process. The first step will involve screening, extracting data and charting data from literature identified through the initial combined systematic search. The second stage will involve refining the framework and building theory. This will be achieved through identifying and coding any additional relevant data that adds to the framework in papers screened for inclusion in the systematic review, as well as exploratory searching using terms related to newly identified concepts and theoretical sampling of studies from this wider literature to develop and elaborate on emerging themes from the analysis [28]. We will focus on reaching theoretical saturation [29] (the point at which no new themes are emerging from the literature) rather than trying to capture the literature in its entirety. Our aim will be to develop ‘good enough’ [15] constructs to inform theory to explain the mechanisms through which the different features of weekend as opposed to weekday care are likely to have an impact, as well as the potential contextual modifiers. We will conduct a further two to four focus groups with staff and patients to develop and amend the framework. We will draw on theories and models from other fields including patient safety, to make sense of the findings from the synthesis, and will also consult with expert members of our advisory group to aid interpretation.

### Integrating findings from the two component reviews

Findings from the framework synthesis will be considered in relation to findings from the conventional systematic review. For example, where a potential moderator for the weekend effect is identified from the meta-regression, the finding will be reflected upon to see if it is compatible with the logic model under development. Similarly, preliminary findings from framework synthesis may provide hypotheses that could be tested through further exploratory quantitative analyses to help explain heterogeneity observed between studies included in the systematic review. In addition to the continuous modification of the preliminary logic model based on findings from the second stage of the framework synthesis and focus groups as described above, a workshop session bringing together key individuals involved in each review and an iterative process of discussion and collaborative writing will also be anchored to further modification of the logic model, which in turn will be used to inform two complementary publications from the two components of the review.

### Amendments to the protocol

Any amendments to the protocol will be documented. Records in the PROSPERO will be updated when important changes related to study selection, risk of bias assessment and/or data analysis are introduced. All deviations from the protocol will be described when the review is written up for publication, along with rationale behind the changes.

## Discussion

This mixed methods review aims to quantify the magnitude of the weekend effect as well as provide greater insight into the mechanisms behind this complex phenomenon. Although a number of literature reviews have been conducted on related topics, they have either focused on specific disease conditions (e.g. myocardial infarction [8] and stroke [9]) or setting (e.g. intensive care units [10]) or adopted a ‘rapid review’ approach without detailed critical appraisal and quantitative synthesis of finding of individual studies [1, 2]. More importantly, an effort that systematically explore both the magnitude and possible mechanisms of the weekend effect through careful examination of diverse evidence, both quantitative and qualitative, appears to be lacking. Given the fast growing literature on this topic and the substantial public attention it has attracted, our proposed review is timely and will provide the best evidence to inform relevant debate and policy makers.

There are novel features in the mixed methods review that we propose here. First, given the complexity of the topic, we did not start with a rapid scoping search that is commonly adopted during the planning of a review. Instead, we undertook a comprehensive search using a broad and inclusive strategy to comprehensively capture potentially relevant studies, followed by an iterative and detailed screening process to gauge both the volume and nature of available evidence. This process, although time consuming, gives us a much better appreciation of the literature and allows us to prioritise our focus among the vast literature and to optimise the areas covered by the two components of the review. Second, the framework of the proposed review is informed not only by the expertise of the review team and the initial exploration of the literature, but also by focus groups that gathered the views of patients and health care providers. A similar, iterative process will be followed during the preparation of the review, providing further opportunities for wider participation and input from relevant stakeholders. This will help ensure the validity and relevance of the review methods and findings. Third, this review is undertaken alongside the five-year long HiSLAC project, which will generate further data on the magnitude and mechanisms of the weekend effect. The findings of the review can be updated when the new data become available and therefore will enable the new evidence to be incorporated into existing evidence and be interpreted in the context of the totality of available evidence. While this has long been advocated as the model for evidence based medicine and examples are growing for clinical trials [30], our adoption of this approach is relatively novel for observational health services research.

There are limitations to the planned systematic review. The volume of potentially relevant literature is large, and we have had to make a decision to focus on undifferentiated hospital admissions for the quantitative synthesis of weekend effect to ensure feasibility. Nevertheless, our detailed screening of records retrieved from our search will provide a comprehensive list of relevant studies on admissions related to specific conditions, which can be utilised for further systematic reviews in the future.

The topic of the weekend effect has attracted substantial attention among clinicians, academics and the general public. During our scoping and preparation of this protocol, we became aware of some concurrent work and expressed intention to undertaking review of literature in related fields [11, 31–34]. While we believe our proposed review is the most comprehensive to date, a view confirmed by the advice of the HiSLAC project’s steering committee, we will be open to collaboration with other researchers to maximise knowledge gained through this effort and minimise potential duplication of work.

### Ethics approval and consent to participate

Not applicable

### Consent for publication

Not applicable

## Additional files

Additional file 1: PRISMA-P 2015 checklist. (DOCX 30 kb) Additional file 2: Appendices: Appendix 1 Search strategy for MEDLINE & Appendix 2 Study screening form. (PDF 355 kb)

## Abbreviations

CINAHL: Cumulative Index to Nursing and Allied Health Literature
CPCI: Conference Proceedings Citation index
EthOS: e-theses Online Service
FFT: Family and Friend Test
GRADE: Grading of Recommendations Assessment, Development and Evaluation
HiSLAC: High-intensity Specialist Led Acute Care
HMIC: Health Management Information Consortium
MOOSE: Meta-analysis Of Observational Studies in Epidemiology
NHS: National Health Service
PRISMA-P: Preferred Reporting Items for Systematic Review and Meta-Analysis Protocols
UK: United Kingdom
WAIC: Watanabe Akaike Information Criterion
