# Supplementary material for: The magnitude and mechanisms of the weekend effect in hospital admissions: A protocol for a mixed methods review incorporating a systematic review and framework synthesis
Source: Syst Rev. 2016 May 21;5:84. doi: 10.1186/s13643-016-0260-2 (PMC4875695; doi:10.1186/s13643-016-0260-2)
Supplement: Additional file 2: — Appendices: Appendix 1 Search strategy for MEDLINE & Appendix 2 Study screening form. (PDF 355 kb) [file 13643_2016_260_MOESM2_ESM.pdf]

The magnitude and mechanisms of the weekend effect in hospital admissions: A protocol for a mixed method review incorporating a systematic review and framework synthesis

The HiSLAC Collaboration

Appendices

Appendix 1 Search strategy for MEDLINE

1. MEDLINE; weeken*.ti,ab; 5300 results.
2. MEDLINE; "week en*".ti,ab; 956 results.
3. MEDLINE; "out of hour*".ti,ab; 1378 results.
4. MEDLINE; exp AFTER-HOURS CARE/; 1118 results.
5. MEDLINE; "after hours".ti,ab; 883 results.
6. MEDLINE; weekday*.ti,ab; 2909 results.
7. MEDLINE; "week day*".ti,ab; 315 results.
8. MEDLINE; exp PATIENT ADMISSION/; 19058 results.
9. MEDLINE; exp HOSPITALIZATION/; 165683 results.
10. MEDLINE; exp PATIENT DISCHARGE/; 19783 results.
11. MEDLINE; exp EMERGENCY SERVICE, HOSPITAL/; 51511 results.
12. MEDLINE; exp EMERGENCY MEDICAL SERVICES/; 98612 results.
13. MEDLINE; admission*.ti,ab; 144697 results.
14. MEDLINE; discharge*.ti,ab; 174549 results.
15. MEDLINE; exp SECONDARY CARE/; 143 results.
16. MEDLINE; "secondary care".ti,ab; 3640 results.
17. MEDLINE; hospital*.ti,ab; 871513 results.
18. MEDLINE; 1 OR 2 OR 3 OR 4 OR 5 OR 6 OR 7; 10395 results.
19. MEDLINE; 8 OR 9 OR 10 OR 11 OR 12 OR 13 OR 14 OR 15 OR 16 OR 17; 1135206 results.
20. MEDLINE; 18 AND 19; 3211 results.
21. MEDLINE; 20 [Limit to: Humans and Publication Year 2000-2015]; 2173 results

Appendix 2 Study screening form

HiSLAC Literature Review 2nd Round Screening Form

Instructions

Records that have passed the 1st round of screening were considered potentially relevant to weekend effects. These have been broadly grouped into four types of studies (1)-(4) as shown in Figure below. The main purpose for this (2nd) round of screening is to make a near-final* judgement on the eligibility of each paper according to the updated inclusion/exclusion criteria and to assign each record to one of the relevant destinations within the literature review: systematic review (SR), systematic review appendix (SRAX), framework synthesis (FS), framework synthesis appendix (FSAX) or exclude (EX) based on the coding scheme shown below and overleaf.

*The final decision could still change upon examination of full-texts or subsequent adjustment of inclusion criteria for framework synthesis (which is meant to be an iterative process with some flexibility regarding the literature to be consulted and included)

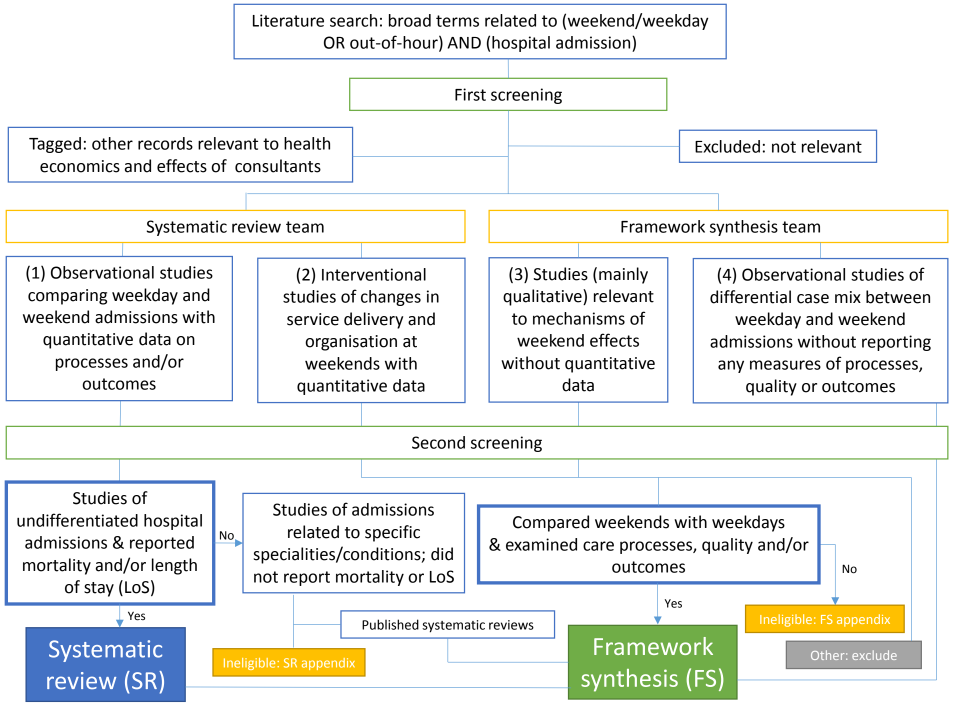

Systematic review team – further coding of type (1) studies from 1st screening

Criteria	Details and examples	Action and coding for '2nd screening'
**1. Absence of other reasons for exclusion**	Note: this item is mainly to eliminate less relevant records that were retained from the 1st screening due to insufficient information or 'just in case'	
Yes		Proceed to criterion 2
No	Examples of studies to be excluded<br>**Comparison**: did not compare weekends with weekdays; only compared night time with day night<br>**Study design**: narrative review, commentary, editorial, letters without presenting original data<br>**Setting**: long-term care facilities, primary care, primary care out-of-hour services, community services, dental services	Exclude;<br>Code EX for exclusion or BK if it could be very useful background information; provide comments as needed
**2. Undifferentiated admissions**		
Yes	All admissions; all medical admissions; all surgical admissions; all emergency admissions; all elective admissions; all adult admissions; all paediatric admissions	Proceed to criterion 3
No	Admissions for specific diagnostic groups, conditions or specialities; specialist hospitals or units, e.g. maternity hospitals, mental health hospitals, intensive care unit (ICU), stroke centre, trauma centre, cancer unit; patients of selected age groups<br><br>**Further action**<br>Systematic reviews of selected admissions listed above	Tag for systematic review appendix – exclude due to **S**elected admissions<br>Code SRAX_S<br><br>Refer to framework synthesis team
**3. Reported outcome of interest**		
Yes	Mortality; length of stay	Include in systematic review<br>Code SR
No	Only reported outcomes other than mortality and length of stay (e.g. only reported measures of clinical processes)<br><br>**Further action**<br>Studies that reported information on costs	Tag for systematic review appendix – exclude due to **O**utcomes<br>Code SRAX_O<br><br>Refer to health economics team

Framework synthesis team – further coding of type (2) & (3) studies from 1st screening

Criteria	Details and examples	Action and coding for '2nd screening'
**1. Absence of other reasons for exclusion**	Note: this item is mainly to eliminate less relevant records that were retained from the 1st screening due to insufficient information or 'just in case'	
Yes		Proceed to criterion 2
No	Examples of studies to be excluded<br>**Comparison**: only compared night time with day night<br>**Study design**: narrative review, commentary, editorial, letters without presenting original data<br>**Setting**: long-term care facilities, primary care, primary care out-of-hour services, community services, dental services	Exclude;<br>Code EX for exclusion or BK if it could be very useful background information; provide comments as needed
**2. Compared weekends with weekdays**		
Yes		Proceed to criterion 3
No	Examples<br>Studies that looked at care quality, outcomes or experience at weekends without comparing them to weekdays	Tag for framework synthesis appendix – exclude due to lack of **C**omparison<br>Code **FSAX_C**
**3. Described care quality or outcomes of interest**		
Yes	Studies that described care quality (as defined by study author; including errors) and/or outcomes (including adverse events, staff, carer and patient experiences, quality of life)	Include in framework synthesis<br>Code **FS**
No	Examples<br>Studies that only described service provision/ utilization pattern without having also examined care quality or outcomes; e.g. described differences in bed usage or staff/service availability between weekdays and weekends without exploring its impact on care quality and/or outcomes	Tag for framework synthesis appendix – exclude due to lack of **O**utcomes<br>Code **FSAX_O**
